# Mutation of the Conserved Threonine 8 within the Human ARF Tumour Suppressor Protein Regulates Autophagy

**DOI:** 10.3390/biom12010126

**Published:** 2022-01-13

**Authors:** Rosa Fontana, Daniela Guidone, Tiziana Angrisano, Viola Calabrò, Alessandra Pollice, Girolama La Mantia, Maria Vivo

**Affiliations:** 1 Department of Biology, University of Naples Federico II, 80126 Naples, Italy; rofontana@ucsd.edu (R.F.); d.guidone@tigem.it (D.G.); tangrisa@unina.it (T.A.); vcalabro@unina.it (V.C.); apollice@unina.it (A.P.); lamantia@unina.it (G.L.M.); 2 Department of Chemistry and Biology “Adolfo Zambelli”, University of Salerno, Via Giovanni Paolo II 132, 84084 Fisciano, Italy

**Keywords:** INK4a/ARF locus, autophagy, cancer, LC3, cytoskeleton

## Abstract

Background: The ARF tumour suppressor plays a well-established role as a tumour suppressor, halting cell growth by both p53-dependent and independent pathways in several cellular stress response circuits. However, data collected in recent years challenged the traditional role of this protein as a tumour suppressor. Cancer cells expressing high ARF levels showed that its expression, far from being dispensable, is required to guarantee tumour cell survival. In particular, ARF can promote autophagy, a self-digestion pathway that helps cells cope with stressful growth conditions arising during both physiological and pathological processes. Methods: We previously showed that ARF is regulated through the activation of the protein kinase C (PKC)-dependent pathway and that an ARF phospho-mimetic mutant on the threonine residue 8, ARF-T8D, sustains cell proliferation in HeLa cells. We now explored the role of ARF phosphorylation in both basal and starvation-induced autophagy by analysing autophagic flux in cells transfected with either WT and ARF phosphorylation mutants by immunoblot and immunofluorescence. Results: Here, we show that endogenous ARF expression in HeLa cells is required for starvation-induced autophagy. Further, we provide evidence that the hyper-expression of ARF-T8D appears to inhibit autophagy in both HeLa and lung cancer cells H1299. This effect is due to the cells’ inability to elicit autophagosomes formation upon T8D expression. Conclusions: Our results lead to the hypothesis that ARF phosphorylation could be a mechanism through which the protein promotes or counteracts autophagy. Several observations underline how autophagy could serve a dual role in cancer progression, either protecting healthy cells from damage or aiding cancerous cells to survive. Our results indicate that ARF phosphorylation controls protein’s ability to promote or counteract autophagy, providing evidence of the dual role played by ARF in cancer progression.

## 1. Introduction

One of the most well-defined functions of the tumour suppressor p14ARF, encoded by the alternative reading frame of the CDKN2a locus, is the suppression of aberrant cell growth in response to oncogenes’ activation or hyper-proliferative stimuli [1,2,3,4]. In particular, ARF activates the transcription factor p53 that triggers the expression of apoptosis inducers and cell cycle inhibitory genes. This function is accomplished through the inhibition of the functions of the MDM2 oncoprotein (mouse double minute 2, HDM2 in human), thus allowing p53 stabilization and the activation of downstream pathways [5]. ARF is a highly basic (>20% arginine content) and hydrophobic polypeptide of 132 amino acids (14 kDa) able to regulate cell cycle arrest and/or apoptosis by both p53-dependent and independent pathways. Interestingly, the ARF N-terminal region is necessary and sufficient to fulfil almost all of the known ARF tumour suppressor functions [3].

So far it has been shown how p14ARF exerts a broader role in cell protection as, in addition to oncogenic stimuli, it can respond to a variety of different stresses arising in both pathological and physiological conditions, such as development, differentiation, aging and autophagy [6,7,8,9]. In particular, although since its discovery ARF was linked to anti-oncogenic functions, several experimental observations suggested for the first time that ARF can promote tumour progression [3]. In many cases, tumours retaining ARF expression evolve to metastatic and invasive phenotypes and are associated with a poor prognosis in humans [10,11,12]. ARF involvement in tumourigenesis is at least in part linked to its ability to induce autophagy [13]. Autophagy is a cellular pathway that upon stressful growth conditions or damage recycles proteins or organelles within the cells. Although the role in autophagy has been attributed to a shorter form of ARF protein called smARF [14], it has been now also extensively shown that full-length ARF proteins (both human and mouse) can promote autophagy in a p53-dependent and independent manner [8,9] and in particular that the domain required for autophagy induction resides within the C-terminal domain of the protein [15]. We previously showed that p14ARF levels are regulated by a protein kinase C (PKC)-dependent mechanism [16,17]. The PKC plays an important role in a number of cell functions. We observed that, while the expression of un-phosphorylatable status of the protein on threonine 8 (T8A mutant) does not affect the ability to restrain cell proliferation, the T8D ARF mutant, which corresponds to the constitutive phosphorylated status of the protein, confers growth advantage to HeLa cells, leading to the hypothesis that ARF function might be regulated by phosphorylation on this conserved residue. These data prompted us to explore the hypothesis that ARF phosphorylation could also regulate its role in autophagy. We now provide evidence that in HeLa cells expressing low but detectable ARF levels, the endogenous protein is required for both starvation-induced and basal autophagy. In contrast, endogenous ARF is dispensable in H1299 lung cancer cells. Furthermore, the expression of ARF mutant T8D negatively regulates autophagy in both cell lines, thus suggesting that phosphorylation of this residue is critically involved in the regulation of autophagy.

## 2. Materials and Methods

Cell cultures, transfections and plasmids. HeLa and H1299 cells were purchased from Cell Line Service (CLS, Germany) and cultured in DMEM supplemented with 10% FBS at 37 °C and 5% CO_2_ as described in [18]. pcDNA3.1 ARF and T8 mutant plasmids were previously described [17]. Transfection was performed with Lipofectamine 2000 (Thermo Fisher, Waltham, MA, USA) as described previously. Where indicated, cells were starved by incubating cells with Hanks buffered salt solution (HBSS, from Thermo Fisher, Waltham, MA, USA) with or without chloroquine at 50 μM final concentration for 2.5 h.

For RNA interference, p14ARF siRNA and control scrambled siRNA were previously described in [19,20]. Briefly, cells were seeded at 60% confluence (2.5 × 10^5^ cells/well) in 60 mm dishes and transiently transfected with RNAiMAx (Thermo Fisher, Waltham, MA, USA) according to the manufacturer’s recommendations. p14ARF siRNA or scrambled siRNA were used at 10 mM.

Immunoblotting, immunofluorescence and antibodies. Western blot (WB) analysis was performed as previously described. We used anti-ARF (C-18) and actin (I-19) (Santa Cruz Biotechnology, Dallas, TX, USA) and anti-LC3 (rabbit polyclonal, Novus Biological, Milano, Italy). Images were acquired by a ChemiDoc Imaging System (Bio-Rad, Segrate (MI), Italy). Representative experiments are shown for each blot. Band intensities were quantified by ImageJ software (https://imagej.net/software/fiji/, version 2.0.0-rc-54/1.51h, Date: 8 September 2016 T11), normalized respect loading control and reported as fold enrichment with respect to the control sample. For IF experiments, cells were treated as described in [19,20,21] with anti-X-press antibody (Thermo Scientific, Thermo Fisher, Waltham, MA, USA) followed by anti-mouse Cy3 conjugated secondary antibody. Images were taken with a Zeiss (Oberkochen, Germany) confocal laser-scanning microscope Axio Observer. A 40× objective was used, and image analysis was performed using ImageJ. Live phase-contrast images were acquired using a Nikon Eclipse microscope (Nikon, Tokyo, Japan) [19]. All data are expressed as the means of independent experiments (biological replicates) with standard deviations (SD). Analysis of variance was performed by the Student’s *T*-test as described in [22].

## 3. Results

### 3.1. ARF Silencing Decreases Autophagic Flux in HeLa Cells

**Figure 1:**
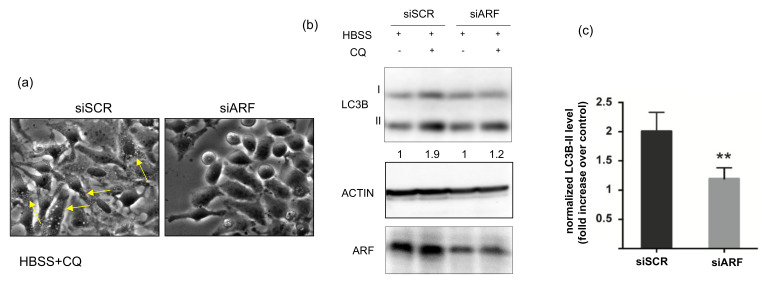
Effect of ARF silencing on autophagy upon starvation. HeLa cells treated with the indicated siRNAs were incubated in Hanks buffered salt solution (HBSS) to induce amino acid and growth factors starvation and with 50 µM chloroquine (CQ) to inhibit lysosome-mediated degradation. Living cells were analysed by phase-contrast microscopy (**a**) and Western blot (**b**) to measure LC3 lipidation (LC3I to LC3II conversion). Actin is a loading control. ARF Western shows the silencing efficiency. LC3B-II levels normalized vs. actin are shown as fold increase relative to CQ^−^ cells (arbitrarily set to 1) below the Western. Graph on the right-hand side (**c**) represents the quantification of normalized LC3 levels from three independent replicates performed with ImageJ. Error bars indicate SD, *n* = 3. ** *p* < 0.05.

To analyse the role of ARF in autophagy, we first determined the involvement of the endogenously expressed protein in starvation-induced autophagy in HeLa cells. To this aim, we downregulated ARF by RNA interference and compared the autophagic flux by Western blot in control and silenced samples. To induce autophagy, we incubated cells with the amino acid-free medium HBSS. Upon autophagy induction, the protein LC3-I is recruited on the phagophore membrane and converted to its lipidated form, LC3-II, which induces its growth and its closure in the mature autophagosome. As upon autophagosome fusion with the lysosome, lipidated LC3 (LC3-II) is degraded together with the cargo, to quantify the whole cellular pool, and thus quantify the autophagic flux, cells are usually incubated with an inhibitor of the proteolytic activity of lysosomal enzymes. To this aim, forty-eight hours post-transfection control (siSCR) or ARF-specific siRNA (siARF)-treated cells were starved with HBSS and treated or not with the protease inhibitor chloroquine (CQ). Cells were first observed by phase-contrast microscopy to detect autophagosome formation, and subsequently, cellular extracts were prepared and analysed. While control cells displayed well visible granules within the cytoplasm resembling autophagosomes (Figure 1a, yellow arrows), ARF-silenced cells failed to do so. Immunoblot analysis show that the amount of LC3-II in ARF silenced cells was reduced to 50% compared with control cells, showing that the autophagic pathway was impaired, in line with previous data in other cell contexts [13,15]. We next analysed the effect of ARF endogenous expression on basal autophagy in these cells. The experiments showed that ARF decrease was accompanied by a decreased autophagic flux, although to a lesser extent than upon starvation (data not shown). Collectively these experiments show us that ARF is required in HeLa cells to induce autophagy.

### 3.2. Mimicking ARF Phosphorylation Impairs Autophagy in Both HeLa and H1299 Cells

**Figure 2:**
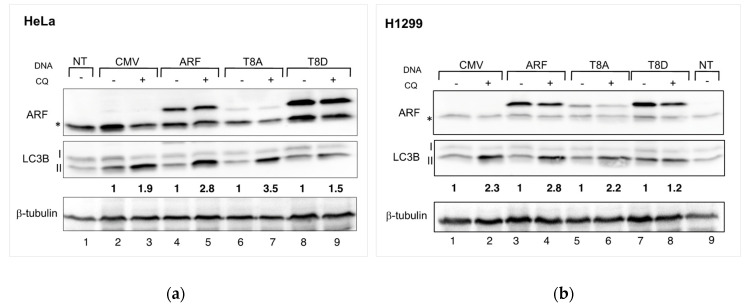
(**a**) HeLa cells, transfected with the indicated plasmids, were incubated with 50 µM CQ twenty-four hours after transfection, as described in Materials and Methods. Cell extracts were subjected to SDS-page and incubated with anti-ARF to check exogenous expression, tubulin as a loading control and anti-LC3 to measure the autophagic flux. (**b**) H1299 cells treated as in (**a**). Densitometric analysis of LC3B-II levels were normalized vs. tubulin and in CQ treated samples are expressed as fold increase relative to LC3B-II level in untreated cells, in which it has been arbitrarily set to 1. The so obtained autophagic flux is reported below the Western for each transfection. Densitometric analysis was performed with Fiji (See Materials and Methods for details). Representative images of three independent experiments are shown. Asterisks indicate endogenous expressed ARF.

In our past experiments, we observed that ARF T8D expression increases cell proliferation in HeLa cells but does not affect H1299 cells [16]. Remarkably, we demonstrated that such residue, lying within a consensus for PKC, can be phosphorylated in vivo [17]. Given that ARF appears to be required in HeLa cells to induce autophagy efficiently, we analysed whether threonine at position 8 was involved in ARF’s ability to induce autophagy. Thus, we used both HeLa and H1299 cells to determine the effect of WT and mutant ARF expression on the autophagic flux. Given that autophagy pathways depend on the cooperation among several players, both upstream and downstream ARF, we decided to look at the level of lipidated LC3 during basal autophagy. We thus transfected cells with plasmids encoding either WT or T8 mutants, treated them with CQ for 2.5 h and performed Western blots of cellular extracts. The autophagic flux is estimated as the increment of the lipidated form of LC3B (LC3B-II) obtained upon CQ treatment. Densitometric analysis of immunoblots showed that, in contrast to the WT and T8A transfections, T8D-expressing cells are insensitive to CQ treatment (Figure 2 left-hand side, compare lane 9 vs. lane 8 and densitometric analysis below the Western). Similar experiments performed in H1299 cells gave similar results (Figure 2, right-hand side; compare lane 8 vs. 7 in H1299 cells).

We next analysed the autophagic pathway, checking the protein levels of the long-lived autophagic marker p62/SQSTM1. This protein encodes a cargo adaptor protein that interacts with autophagic substrates and delivers them to the autophagosome for degradation. In this process, p62 is degraded, so if autophagy is inhibited, it accumulates in the cell. We therefore compared p62 protein levels in mock and ARF or T8D transfected cells. The experiment showed an increased level of p62 in T8D-expressing cells, thus indicating an impaired autophagic flux (Supplementary Figure S1). These experiments thus collectively suggest that expression of T8D ARF mutant is associated with a decreased autophagic flux. 

### 3.3. ARF T8D Expression Impairs Autophagosomes Formation in HeLa Cells

**Figure 3:**
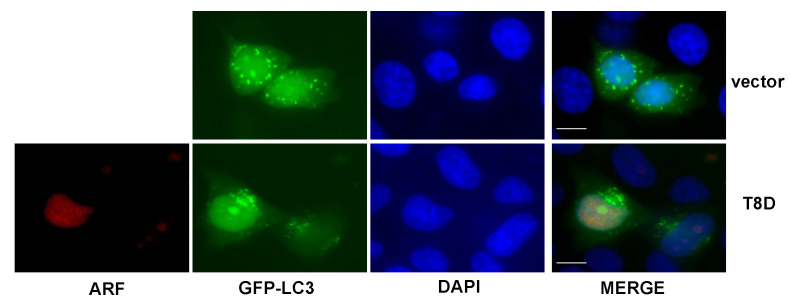
HeLa cells were transfected with GFP-tagged LC3 protein-expressing plasmid, with or without ARF-T8D-expressing vector. Twenty-four hours post-transfection, cells were incubated in HBSS and 50 µM chloroquine to visualize autophagosomes formation. ARF was visualized using an anti-X-press antibody and nuclei by DAPI staining. Representative images were taken with a Zeiss confocal laser-scanning microscope, as previously described (see Section 2). Scale bar 10 μm.

To get further insights into the mechanisms of T8D inhibition of autophagy, we used a GFP tagged LC3 expression plasmid and analysed the formation of autophagosomes in HeLa cells transfected with a T8D expression construct by fluorescence microscopy. To visualise the GFP-LC3II-loaded autophagosomes, cells were starved twenty-four hours after transfection with HBSS and incubated with 50 µM chloroquine. The experiment shows that, while control vector-transfected cells clearly display GFP fluorescent granules, T8D transfected cells show a diffuse tagged LC3 localisation within the cells (Figure 3), thus suggesting that in the presence of ARF T8D, an impairment in autophagosomes formation can be the cause of the previously observed decreased autophagic flux.

## 4. Discussion

We already showed in previously published studies that p14ARF can be regulated by PKC-dependent mechanisms in cancer cells. Several PKC target sites have been identified in the sequence of p14ARF, and among these, the threonine 8 residue is highly conserved [17]. While mimicking the un-phosphorylatable status of the protein on threonine 8 (T8A mutant) does not affect ARF ability to restrain cell proliferation, the opposite mutation in the T8D ARF mutant confers a proliferative advantage when overexpressed in HeLa cells. Several studies reported that p14ARF plays an established role in autophagy, a survival mechanism that can be used by cells to cope with stress arising during cancer evolution. As ARF involvement in autophagy appears cell type-specific, we analysed the effect of ARF downregulation in HeLa cells by RNA-mediated silencing. We found that decreasing ARF levels were correlated with decreased autophagic flux. This effect could be caused by a decrease in ARF protein actively involved in autophagy or to the inhibitory effect of the stable (phosphorylated) ARF protein that persists after ARF silencing. We thus focused our attention on the role of threonine 8 phosphorylation in this pathway. In particular, in this study, we analysed the role of ARF protein in autophagy in two cancer cell lines where ARF silencing has a different outcome in terms of cell proliferation control. While in HeLa cells ARF T8D expression confers a growth advantage, in lung cancerous H1299 cells, it displays no effect. In contrast, in both cell lines, the expression of ARF T8D inhibits basal autophagy with a reduction in autophagosomes and an accumulation of LC3 protein within the nucleus. T8A expression increases the autophagic flux with respect to the mock-transfected cells (Figure 2); however, given its lower stability, we cannot rule out the possibility that this mutation impairs autophagy as well, and further experiments need to be carried out to clarify this point. In both cases, the key role of this residue in autophagy regulation still remains.

It has been widely shown that ARF tumour suppressor ability mainly resides within the exon-1-encoded domain [23,24,25]. Although early evidence led to the identification of a shorter ARF isoform smARF (small ARF) that accounted for its involvement in autophagy, it has been shown that the full-length ARF proteins (both human and mouse) promote autophagy in both a p53-dependent and independent manner [26]. Recent observation showed that the ARF C-terminal domain, and in particular the region between amino acids 100 to 120, is necessary for autophagy induction by both human and murine protein [15]. The different roles of ARF T8 mutants in autophagy led us to the hypothesis that post-translational modifications of the N-terminal ARF region on threonine 8, or modification in the protein stability, could be required to activate or deactivate the function of exon-2-encoded domain. Additionally, the exon-2-encoded C-terminal domain is required for ARF protection from anoikis [19]. To date, the majority of studies on ARF has focused on its tumour suppressor roles. However, new pieces of evidence are paving the way to the hypothesis that ARF might promote survival [3]. We and others found that, in some cellular contexts, the protein exerts a protective role within the cell. This aspect is exacerbated in some tumours such as muscular-invasive bladder cancer and in prostate cancers, where not only ARF levels appear increased, but the protein is also involved in the epithelial-to-mesenchymal transition and chemoresistance [10,11,12]. In light of this evidence, it is crucial to better define the molecular mechanisms at the basis of ARF involvement in cancer evolution. Taken together, our observations strongly suggest that, while the N-terminal domain of the protein is involved in tumour suppression, the C-terminal domain could be instead responsible for the pro-proliferative functions of the protein.

## Data Availability

The data presented in this study are available in the main text, figures, tables and Supplementary Material.

## Supplementary Materials

The following supporting information can be downloaded at: https://www.mdpi.com/article/10.3390/biom12010126/s1, Figure S1: HeLa cells were transfected with the indicated plasmids. Cell extracts were subjected to SDS-page and incubated with anti-ARF antibody to check exogenous expression, tubulin as a loading control and anti p62 as autophagic marker. Representative images of three independent experiments are shown.

## References

[1] Sharpless N.E. (2005). INK4a/ARF: A multifunctional tumour suppressor locus. Mutat. Res..

[2] Ozenne P., Eymin B., Brambilla E., Gazzeri S. (2010). The ARF tumour suppressor: Structure, functions and status in cancer. Int. J. Cancer.

[3] Fontana R., Ranieri M., La Mantia G., Vivo M. (2019). Dual Role of the Alternative Reading Frame ARF Protein in Cancer. Biomolecules.

[4] Zindy F., Williams R.T., Baudino T.A., Rehg J.E., Skapek S.X., Cleveland J.L., Roussel M.F., Sherr C.J. (2003). Arf tumour suppressor promoter monitors latent oncogenic signals in vivo. Proc. Natl. Acad. Sci. USA.

[5] Sherr C.J., Weber J.D. (2000). The ARF/p53 pathway. Curr. Opin. Genet. Dev..

[6] Kotsinas A., Papanagnou P., Evangelou K., Trigas G.C., Kostourou V., Townsend P., Gorgoulis V.G. (2014). ARF: A versatile DNA damage response ally at the crossroads of development and tumourigenesis. Front. Genet..

[7] Sherr C.J. (2006). Autophagy by ARF: A short story. Mol. Cell.

[8] Fontana R., Vivo M. (2018). Dynamics of p14ARF and Focal Adhesion Kinase-Mediated Autophagy in Cancer. Cancers.

[9] Pimkina J., Humbey O., Zilfou J.T., Jarnik M., Murphy M.E. (2009). ARF induces autophagy by virtue of interaction with Bcl-xl. J. Biol. Chem..

[10] Owczarek T.B., Kobayashi T., Ramirez R., Rong L., Puzio-Kuter A.M., Iyer G., Teo M.Y., Sanchez-Vega F., Wang J., Schultz N. (2017). ARF Confers a Context-Dependent Response to Chemotherapy in Muscle-Invasive Bladder Cancer. Cancer Res..

[11] Ferru A., Fromont G., Gibelin H., Guilhot J., Savagner F., Tourani J.M., Kraimps J.L., Larsen C.J., Karayan-Tapon L. (2006). The status of CDKN2A alpha (p16INK4A) and beta (p14ARF) transcripts in thyroid tumour progression. Br. J. Cancer.

[12] Chen Z., Carracedo A., Lin H.K., Koutcher J.A., Behrendt N., Egia A., Alimonti A., Carver B.S., Gerald W., Teruya-Feldstein J. (2009). Differential p53-independent outcomes of p19(Arf) loss in oncogenesis. Sci. Signal..

[13] Humbey O., Pimkina J., Zilfou J.T., Jarnik M., Dominguez-Brauer C., Burgess D.J., Eischen C.M., Murphy M.E. (2008). The ARF tumour suppressor can promote the progression of some tumours. Cancer Res..

[14] Reef S., Zalckvar E., Shifman O., Bialik S., Sabanay H., Oren M., Kimchi A. (2006). A short mitochondrial form of p19ARF induces autophagy and caspase-independent cell death. Mol. Cell.

[15] Budina-Kolomets A., Hontz R.D., Pimkina J., Murphy M.E. (2013). A conserved domain in exon 2 coding for the human and murine ARF tumour suppressor protein is required for autophagy induction. Autophagy.

[16] Fontana R., Guidone D., Sangermano F., Calabro V., Pollice A., La Mantia G., Vivo M. (2018). PKC Dependent p14ARF Phosphorylation on Threonine 8 Drives Cell Proliferation. Sci. Rep..

[17] Vivo M., Ranieri M., Sansone F., Santoriello C., Calogero R.A., Calabro V., Pollice A., La Mantia G. (2013). Mimicking p14ARF phosphorylation influences its ability to restrain cell proliferation. PLoS ONE.

[18] Di Costanzo A., Festa L., Roscigno G., Vivo M., Pollice A., Morasso M., La Mantia G., Calabro V. (2011). A dominant mutation etiologic for human tricho-dento-osseous syndrome impairs the ability of DLX3 to downregulate DeltaNp63alpha. J. Cell. Physiol..

[19] Vivo M., Fontana R., Ranieri M., Capasso G., Angrisano T., Pollice A., Calabro V., La Mantia G. (2017). p14ARF interacts with the focal adhesion kinase and protects cells from anoikis. Oncogene.

[20] Vivo M., Matarese M., Sepe M., Di Martino R., Festa L., Calabro V., La Mantia G., Pollice A. (2015). MDM2-mediated degradation of p14ARF: A novel mechanism to control ARF levels in cancer cells. PLoS ONE.

[21] Montano E., Vivo M., Guarino A.M., di Martino O., Di Luccia B., Calabro V., Caserta S., Pollice A. (2019). Colloidal Silver Induces Cytoskeleton Reorganization and E-Cadherin Recruitment at Cell-Cell Contacts in HaCaT Cells. Pharmaceuticals.

[22] Montano E., Pollice A., Lucci V., Falco G., Affinito O., La Mantia G., Vivo M., Angrisano T. (2021). Pancreatic Progenitor Commitment Is Marked by an Increase in Ink4a/Arf Expression. Biomolecules.

[23] Sugimoto M., Kuo M.L., Roussel M.F., Sherr C.J. (2003). Nucleolar Arf tumour suppressor inhibits ribosomal RNA processing. Mol. Cell.

[24] Tago K., Chiocca S., Sherr C.J. (2005). Sumoylation induced by the Arf tumour suppressor: A p53-independent function. Proc. Natl. Acad. Sci. USA.

[25] Weber J.D., Kuo M.L., Bothner B., DiGiammarino E.L., Kriwacki R.W., Roussel M.F., Sherr C.J. (2000). Cooperative signals governing ARF-mdm2 interaction and nucleolar localization of the complex. Mol. Cell. Biol..

[26] Abida W.M., Gu W. (2008). p53-Dependent and p53-independent activation of autophagy by ARF. Cancer Res..

