# Supplementary material for: Mutation of the Conserved Threonine 8 within the Human ARF Tumour Suppressor Protein Regulates Autophagy
Source: Biomolecules. 2022 Jan 13;12(1):126. doi: 10.3390/biom12010126 (PMC8773949; doi:10.3390/biom12010126)
Supplement: Supplementary file 1 [file biomolecules-12-00126-s001.zip › biomolecules-1485154-supplementary.pdf]

Supplementary Figure S1

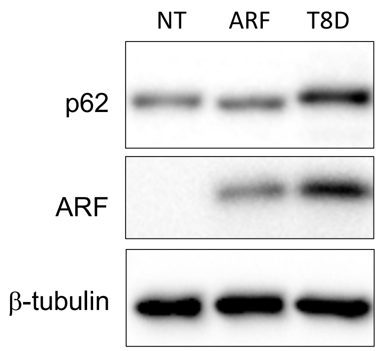

Fig S1. HeLa cells were transfected with the indicated plasmids. Cell extracts were subjected to SDS-page and incubated with anti-ARF antibody to check exogenous expression, tubulin as a loading control and anti p62 as autophagic marker. Representative images of three independent experiments are shown.
